# Volatile Compounds Profile of Sous-Vide Cooked Pork Cheeks as Affected by Cooking Conditions (Vacuum Packaging, Temperature and Time)

**DOI:** 10.3390/molecules181012538

**Published:** 2013-10-10

**Authors:** Jose Sanchez del Pulgar, Mar Roldan, Jorge Ruiz-Carrascal

**Affiliations:** Food Science, School of Veterinary Sciences, University of Extremadura; 10003 Caceres, Spain; E-Mails: jsapuri@hotmail.com (J.S.P.); mar_roldan_romero@hotmail.com (M.R.)

**Keywords:** sous-vide, vacuum cooking, volatile compounds, SPME-GC-MS

## Abstract

The volatile organic compound (VOC) profile of pork cheeks as affected by the cooking conditions was investigated. Pork cheeks were cooked under different combinations of temperature (60 °C or 80 °C), time (5 or 12 h) and vacuum (vacuum or air-packaged). As a general rule, the VOCs originating from lipid degradation were positively affected by the cooking temperature and negatively by the cooking time, reaching the highest amounts in pork cheeks cooked at 80 °C during 5 h and the lowest in samples cooked at 80 °C during 12 h. On the contrary, VOCs originated from amino acids and Maillard reactions were positively affected by both factors. The proportion between lipid degradation and amino acids reactions was estimated by the hexanal/3-methylbutanal ratio, which reached its highest values in samples cooked at 60 °C during 5 h in the presence of air and the lowest values in samples cooked at 80 °C during 12 h, regardless of the vacuum status.

## 1. Introduction

Sous-vide cooking consist on the cooking of raw materials inside heat-stable vacuumized pouches or containers and under controlled conditions of temperature and time, followed by a rapid cooldown to 0–3 °C [1]. This cooking technique has been extensively adopted in the last two decades by catering services and food processing to provide foods of superior sensory quality with a longer shelf-life as compared to those cooked using conventional cook-chill technologies [1,2]. The sous-vide cooking conditions used by chefs for different types of meat are very different to those used for traditional cooking methods or in catering. Thus, commonly recommended combinations of temperature and time by chefs for beef, pork or lamb are around 58–63 °C for 10–48 h, while temperatures for pork in catering most likely reaches 75–80 °C [1]. Only in the last years, a few studies have considered the effects of sous-vide cooking at low temperature for long periods on the physico-chemical changes undergone by meat [3,4,5], but the scientific information available on its effect on the development of cooked meat flavor is scarce.

It is well accepted that raw meat has little or no aroma and a blood-like taste, and that the flavor of cooked meat is thermally developed during cooking, mainly from amino acids and lipids [6]. Quantitatively, lipid oxidation is the major source of volatile compounds in cooked meat, specially the oxidation of unsaturated fatty acids [7]. On the other hand, Strecker degradation of amino acids and Maillard reactions produce a considerable number of branched and heterocyclic volatile compounds with low odor thresholds, which seem to be the main responsibles for the meaty flavor [6,7]. Finally, thiamine degradation generates a series of sulphur-containing compounds with characteristic meaty flavor, such as 2-methyl-3-furanthiol [7,8].

The profile of volatile flavor compounds of cooked meat is strongly affected by the characteristics of the cooking process. Different cooking procedures comprise a wide range of temperatures, from 50 °C in the center of a grilled rare steak, to around 200 °C in its exterior or in the surface of oven-roasted meat, or a constant temperature of around 100 °C during meat stewing. Not only the temperature but also the cooking time and the extent of meat dehydration (*i.e.*, much more intense in the surface of roasted meat than in the stewed meat) are dependent upon the type of cooking [6]. These different cooking conditions affect the type and extent of the chemical reactions leading to the formation of flavor compounds. For example, high temperatures and dehydration enhance Maillard reactions [6], and lipid degradation leads on to different volatile compounds at high temperatures (thermal lipid oxidation) or at moderate to low ones (lipid autoxidation) [7]. Although many studies since the 1950s have focused on the profile of volatile compounds of meat cooked following traditional methods, very scarce scientific information is available on the formation of volatile compounds in sous-vide cooked meat at low temperature and for long times.

It seems clear that the absence of oxygen achieved through vacuum packaging leads to a lower extent of lipid oxidation during the storage of cooked meat and meat products [9]. However, there is not much information about how would it affect the development of the chemical reactions leading to the development of cooked meat flavor during cooking. Previous studies showed lower amounts of hexanal and total amount of volatile compounds in cooked and irradiated vacuum-packaged pork patties as compared to aerobic-packaged ones [10], but no information about the effect of vacuum on the volatile profile of sous-vide cooked meat at low to moderate temperatures is available in the scientific literature. Thus, the purpose of this work was to study the effects of vacuum, cooking temperature and cooking time on the volatile compounds profile of sous-vide cooked pork cheeks.

## 2. Results and Discussion

The headspace solid phase microextraction (SPME) followed by gas chromatography-mass spectrometry (GC-MS) analysis of pork cheeks sous-vide cooked at different time × temperature × vacuum combinations resulted in more than 100 volatile organic compounds (VOCs). This VOCs profile was very similar to that previously described for cooked meat [6,7]. The most representative compounds formed from lipids, amino acids and thiamine were selected, and their average levels (area under the peak curve) in each batch are shown in Table 1.

Selected lipid oxidation VOCs were linear saturated aldehydes (from butanal to nonanal), unsaturated aldehydes (2-decenal and 2,4-decadienal), a ketone (2-octanone), and several furans and furanones (2-methylfuran, 2-ethylfuran, 2-butylfuran, 2-pentylfuran and 5-pentyl-2(5*H*)-furanone). All these compounds have been previously detected in the headspace VOC profile in different types of cooked meats [6,7]. As a general rule, the VOCs derived from fatty acid degradation had a similar trend as affected by time and temperature cooking conditions, with the highest levels in samples cooked at 80 °C during 5 h and the lowest in samples cooked at 80 °C during 12 h. Nevertheless, heptanal, octanal, 2-octanone and 2-methylfuran did not follow such a tendency. These results suggest a promoting effect of heating on the degradation of polyunsaturated fatty acids such as linoleic acid, increasing also the amount of free radicals capable of attacking other fatty acids less susceptible to oxidation, such as oleic acid, leading to the formation of other VOCs like heptanal, octanal, 2-octanone and nonanal in a more advance stage of the lipid oxidation process [11].

**Scheme 1:**
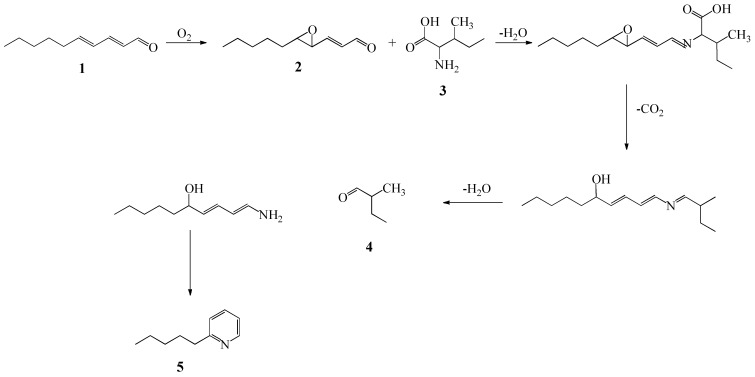
Reaction of 2,4-decadienal with leucine, following [15].

Some of these carbonyl compounds derived from fatty acid oxidation may also react with the amine groups of lysine, cysteine and glutathione [12] and thus, the decrease in their concentration at longer cooking times at higher temperatures might suggest the implication of these compounds on the formation of other VOCs [12,13]. Accordingly, the greatest decrease in the detected levels from 5 to 12 h in samples cooked at 80 °C was found for 2-decenal and 2,4-decadienal, which are known to be more susceptible to further reactions than long chain saturated aldehydes such as octanal [12,13]. In fact, previous studies have shown the reaction of 2,4-decadienal with phenylalanine in model systems through further oxidation of the alkadienal to epoxydecenal [14]. This type of epoxyalkenals has been shown to induce Strecker degradation of amino acids in a similar way to dicarbonyls [15] (Scheme 1).

Selected VOCs derived from degradation of amino acids and/or thiamine, included carbon disulfide, 3-methylbutanal, 1-hydroxy-2-propanone, dimethyl disulfide, 1-(methylthio)-propene, 2-methyl-thiophene, 2-acetyl-2-thiazoline, 2-pentylthiophene and benzothiazole. Contrarily to lipid derived VOCs, some compounds originated through Maillard reactions and/or thiamine degradation, such as 1-hydroxy-2-propanone, dimethyl disulfide, 1-(methylthio)-propene and 2-methylthiophene, were found in samples cooked at 80 °C, while they did not reach detectable levels in samples cooked at 60 °C. Additionally, 3-methylbutanal, an aldehyde formed as a consequence of the Strecker degradation of leucine (Scheme 1), was found at higher concentrations in the samples cooked at 80 °C than in those cooked at 60 °C (Table 1). A similar trend was found in chicken meat cooked to different end-point temperatures [16], although in this latter study samples were cooked for very short times. Therefore, it seems clear that sous-vide cooking of pork cheeks at higher temperature showed a positive influence on the development of volatile compounds from Maillard reactions, and more specifically, from Strecker degradation of amino acids.

**Table 1:** Selected volatile compounds (in area units × 10^−5^) in pork cheeks cooked at different combinations of temperature (60 *vs.* 80 °C), time (5 h *vs.* 12 h) and vacuum (vacuum packaged *vs.* air-packaged).

	LRI^1^	ID^2^	60-5v	60-5a	60-12v	60-12a	80-5v	80-5a	80-12v	80-12a	SEM	P T	P t	P vac	P T × t	P T × vac	P t × vac	P T × t × vac
Carbon disulfide	534	B	22	10	24	10	16	15	25	23	1.81	ns	ns	*	ns	ns	ns	ns
Butanal	593	A	4.61^c^	2.74^c^	5.7^bc^	4.57^c^	10^a^	8.8^ab^	4.19^c^	4.12^c^	0.44	***	**	*	***	ns	ns	ns
2-Methylfuran	605	B	-	-	0.35^†^	-	1.56^ab^	0.90^b^	1.16^ab^	1.84^a^	0.13	-	ns	ns	-	-	**	-
3-Methylbutanal	646	A	3.01^b^	1.35^c^	3.11^b^	2.98^b^	3.17^b^	2.96^b^	4.86^a^	5.60^a^	0.22	***	***	ns	*	*	*	ns
1-Hydroxy-2-propanone	674	B	-	-	-	-	2.26^b^	1.15^b^	15^a^	17^a^	1.28	-	***	ns	-	-	ns	-
Pentanal	797	A	173^a^	111^a^	184^a^	194^a^	385^b^	373^b^	202^a^	173^a^	17	***	**	ns	***	ns	ns	ns
2-Ethylfuran	703	B	5.7^ab^	3.71^b^	6.1^ab^	9.5^ab^	8.3^ab^	15^a^	3.40^b^	7.5^ab^	1.02	ns	ns	ns	*	ns	ns	ns
Dimethyl disulfide	749	A	-	-	-	-	3.88^ab^	2.21^b^	9.7^ab^	12^a^	0.87	-	**	ns	-	-	ns	-
1-(Methylthio)-propane	764	B	-	-	0.42^†^	0.54^†^	2.53	2.58	2.01	2.96	0.23	-	ns	ns	-	-	ns	-
2-Methylthiophene	776	B	-	-	3.25^†^	-	17^ab^	9.5^b^	17^ab^	24^a^	1.64	-	*	ns	-	-	*	-
Hexanal	803	A	2672^ab^	1995^ab^	2583^ab^	2412^ab^	2969^a^	2924^ab^	2052^ab^	1943^b^	92	ns	*	ns	**	ns	ns	ns
2-Butylfuran	894	B	3.61^abc^	2.20^c^	3.32^abc^	3.15^c^	5.6^a^	5.4^ab^	3.09^c^	3.23^bc^	0.24	**	*	ns	**	ns	ns	ns
Heptanal	902	A	271	183	257	299	293	188	263	307	14	ns	ns	ns	ns	ns	**	ns
2-Octanone	992	A	-	-	-	-	6.3^b^	8.5^ab^	8.4^ab^	11^a^	0.75	-	*	*	-	-	ns	-
2-Pentylfuran	994	B	189^b^	120^b^	202^ab^	195^ab^	353^a^	362^a^	141^b^	148^b^	18	**	*	ns	***	ns	ns	ns
Octanal	1004	A	381^ab^	237^b^	345^ab^	278^ab^	482^ab^	400^ab^	434^ab^	519^a^	23	**	ns	ns	ns	ns	ns	ns
Nonanal	1107	A	878	571	784	744	996	1044	729	804	34	*	ns	ns	*	*	ns	ns
2-Acetyl-2-thiazoline	1116	B	1.93	0.77	2.25	1.6	-	-	-	-	0.19	-	ns	*	-	-	ns	-
2-Pentylthiophene	1173	B	1.62	0.37	2.21	0.65	2.34	1.9	0.24	0.3	0.23	ns	ns	*	*	ns	ns	ns
Decenal	1298	A	9.4^ab^	4.81^b^	9.7^ab^	8.3^b^	16^a^	18^a^	4.16^b^	4.91^b^	0.94	ns	**	ns	***	ns	ns	ns
Benzothiazole	1250	B	2.01	1.7	1.89	2.6	2.21	2.16	1.86	1.98	0.1	ns	ns	ns	ns	ns	ns	ns
2,4-Decadienal	1300	A	8.3^ab^	4.47^b^	10^ab^	13^a^	9.4^ab^	14^a^	3.09^b^	4.77^b^	0.79	ns	ns	ns	***	ns	ns	ns
5-Pentyl-2(5 *H*)-furanone	1356	B	4.99^ab^	2.91^ab^	5.2^ab^	6.2^a^	5.0^ab^	5.5^ab^	1.54^b^	2.22^ab^	0.39	*	ns	ns	**	ns	ns	ns

^1^ Linear retention index in the DB-5 column. T: temperature; t: time; vac: vacuum packaging. ^2^ Reliability of identification: A, mass spectrum and retention index identical to those of a standard; B, mass spectrum and retention index in agreement with the corresponding literature data. ^†^ Compound identified in only one sample of the batch. Different superscript letters within the same row mean significant differences between the different temperature × time× vacuum treatments (P < 0.05). ns: No significance. * *p* < 0.05. ** *p* < 0.01. *** *p* < 0.001.

This is no doubt a potential drawback for sous-vide cooked meats at low to moderate temperatures, and in fact, chefs usually heat the meat in an oven or in a grill before serving, to enhance the aroma and color of the surface.

1: 2,4-decadienal; 2: 4,5-epoxy-2-decenal; 3: leucine; 4: 3-methylbutanal; 5: 2-pentylpyridine

In most VOCs from Maillard reactions and from thiamine degradation, with the exception of 1-(methylthio)-propane, cooking time showed a positive effect on the final levels found, which in the case of 2-methylthiophene was only significant in the samples cooked in the presence of air. On the other hand, the effect of cooking time on the final chromatographic areas of 3-methylbutanal was much more evident in samples cooked at 80 °C than in those cooked at 60 °C. These results point out to a positive effect of long cooking times when cooking at moderate to high temperatures on the levels of VOCs originated from amino acids and thiamine, since the involved reactions are favored by the temperature [6], but also seem to be favored by cooking time, since this kind of compounds has been found in long-ripened meat products [17]. In this regard, the Strecker degradation of different amino acids has been shown to strongly depend upon temperature and time [18]. Moreover, higher cooking temperatures and longer cooking times may increase the amount of reactive carbonyls, either from lipids or proteins, which could participate in the initial attack to the amine group of the amino acids [19]. Contrarily, 2-pentylthiophene was higher in samples cooked at 80 °C for 5 h than in those cooked at 80 °C for 12 h (Table 1). This could indicate that the formation of this compound is favored by the temperature, but participates in further reactions and thus, long cooking times may cause a depletion of its levels.

Contrary to the results described for the rest of VOCs originated from degradation of amino acids and/or thiamine, 2-acetyl-2-thiazoline was only found at detectable levels in samples cooked at 60 °C, and it was higher in the vacuum-packaged samples than in air-packaged ones (Table 1). This compound has been pointed out as one of the impact flavor compounds in roasted beef [20], which indicates that cooking favors its formation. However, high temperatures might contribute to further reactions to yield other volatile sulfur compounds, leading to its complete degradation in 60 min at 100 °C [21]. Accordingly, our results suggest that this is not a very stable compound, since moderately high temperatures (80 °C) for long times led to its degradation.

**Figure 1:**
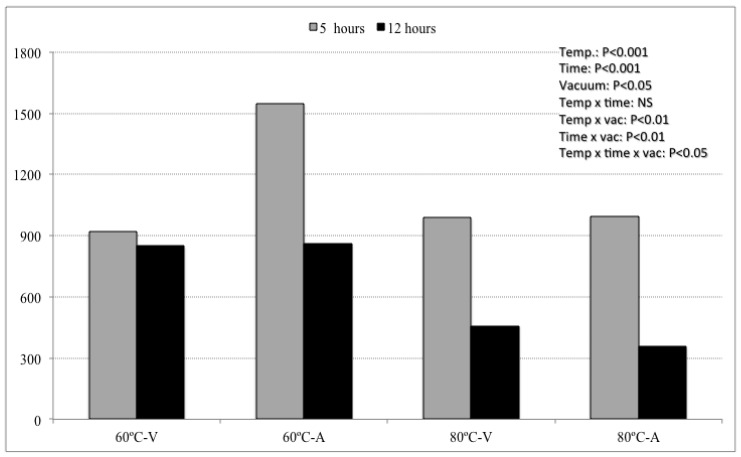
Hexanal/3-methyl butanal ratio in pork cheeks cooked at different combinations of temperature (60 *vs.* 80 °C), time (5 h *vs.* 12 h) and vacuum (vacuum packaged *vs.* air-packaged).

The hexanal/3-methylbutanal ratio has been previously proposed as a way to assess the balance between lipid oxidative reactions and amino acids degradation in meat and meat products [22]. In this study such a ratio was higher in samples cooked at 60 °C than in those cooked at 80 °C, and it was also higher in samples cooked for 12 h than in those cooked for 5 h (Figure 1). This was most likely due to: (1) the positive effect of cooking temperature [6] and time [17] on the generation of compounds due to the degradation of amino acids and (2) to the further reaction of compounds from lipid oxidation with proteins, amino acids and other compounds, which would lead to a decrease on the levels of reactive carbonyls from lipid oxidation [15], as shown in Scheme 1. In addition, this ratio was higher in samples cooked in air-packaged samples than in the vacuum-packaged ones, probably due to the positive effect of the oxygen in the fatty acid oxidation. At last the interaction of the three studied factors had a significantly effect on this ratio, with the highest value in samples cooked at 60 °C during 5 h in the presence of air and the lowest value in samples cooked at 80 °C during 12 h in the presence of air.

## 3. Experimental

### 3.1. Experimental Design

The volatile compounds profile of masseter muscles in Iberian pork cheeks were analyzed when cooked in different time × temperature combinations and when packaged either in the presence of air or under vacuum conditions. The study was carried out using a completely randomized 2 × 2 × 2 design, with eight combinations of time (5 or 12 h), temperature (60 or 80 °C) and vacuum (air-packaged or vacuum-packaged) which were used to cook 40 pork cheeks (n = 5 for each batch). The mentioned combinations of time and temperature were suggested by Chef Antonio Perez (“Atrio” Restaurant, Caceres, Spain). All the samples were from a homogeneous batch of Iberian pigs averaging 150 kg live weight and 15 months age, and were provided by Dehesa de Solana Ltd. (Madrid, Spain) The fresh cheeks presented 73.6% of moisture, 4.3% of fat and 20.7% of protein, with an average weight of 72.8 g, 3.2 cm thickness and 8.1 cm length [4]. The different groups considered in this study and their characteristics are shown in Table 2.

**Table 2:** Temperature, time and vacuum conditions used for cooking the pork cheeks of the different experimental groups considered in the study.

Group	Temperature (°C)	Time (h)	Vacuum
60-5v	60	5	Yes
60-5a	60	5	No
60-12v	60	12	Yes
60-12a	60	12	No
80-5v	80	5	Yes
80-5a	80	5	No
80-12v	80	12	Yes
80-12a	80	12	No

### 3.2. Cooking Procedure

The visible connective and adipose tissue outside of the pork cheeks were removed before packaging at the University of Extremadura's meat pilot plant. Each pork cheek was packaged individually in a plastic bag (nylon/polyethylene pouches; O2 permeability of 9 cm3/m2 per 24 h at 4 °C/80% RH and water steam permeability of 1.2 g/m2 per 24 h; heat resistance of −40 °C/+120 °C) (Joelplas SL, Barcelona, Spain) and heat sealed using a vacuum Tecnotrip EVT-14 packaging machine (Tecnotrip, Barcelona, Spain). The vacuum level was set to 70% of the highest vacuum level allowed by the equipment for the vacuum-packaged batch and to 0% for the non-vacuum-packaged group. The packaged cheeks were then cooked in a thermostatized water bath by applying the time-temperature combinations described above and shown in Table 2.

The temperature of the water in the bath was controlled by using a Testo735-2 thermocouple (Testo, Lenzkirch, Germany). The internal temperature was monitored by using the same thermocouple equipped with a special probe for meat. This device was applied to a different pork cheek that nonetheless had similar characteristics to those of the samples included in each batch. This single sample was not used in the volatile compound profile analysis. After the cooking process finished, the packages were removed from the water bath and submerged in cold water (4 °C) for 30 min. Subsequently, the packaged cheeks were kept under refrigeration (2 °C) overnight. Once the bags were open in order to perform the analyses described in a previous paper [4], a piece of each cheek was taken, vacuum-packaged and kept at −80 °C until volatile compounds analysis.

### 3.3. Volatile Compounds Extraction

Volatile compounds extraction was performed by SPME following a previously developed procedure [23]. After grinding the frozen samples in a coffee mill (Taurus MS 50, Electrodomesticos Taurus, S.L., Lleida, Spain), 5 g of each one were introduced in a 20 mL glass vial, screw-capped with a laminated Teflon-rubber disk. The vial was kept in a water bath at 60 °C for 25 min to equilibrate the volatiles in the headspace. Subsequently, the vial septum was pierced with a SPME needle, and a 2 cm Carboxen/PDMS/DVB fiber (75 µm thickness, Supelco, Bellefonte, PA, USA) was exposed to the headspace for 30 min while the vial was in the 60 °C water bath. Then the fiber was transferred to the gas-chromatograph inlet and desorbed for 15 min at 250 °C (splitless time: 3 min).

### 3.4. Gas Chromatography-Mass Spectrometry

Analyses were performed using an Agilent 6890 series gas chromatograph (Agilent, Avondale, PA USA) coupled to a mass selective detector (Agilent 5973). Analytes were separated using a 5% phenyl-methyl silicone (HP-5) bonded phase fused silica capillary column (Hewlett–Packard, 50 m × 0.32 mm i.d., film thickness 1.05 μm), operating at 45 kPa of column head pressure, resulting in a flow of 1.3 mL/min at 40 °C. The temperature program was 40 °C during 10 min, raised to 200 °C at a rate of 5 °C/min and then raised to 250 °C at a rate of 20 °C/min, maintained at this temperature for 5 min. The transfer line to the mass spectrometer was hold at 280 °C. The mass spectra were obtained by electronic impact at 70 eV, a multiplier voltage of 1756 V and collecting data at a rate of 1 scan/s over the *m/z* range 30–300. *n*-Alkanes (Sigma R-8769, Sigma–Aldrich, Steinhein, Germany) were analyzed under the same conditions to calculate the retention indices (RI) for the volatiles. The compounds were identified by comparison with the mass spectrum and RI of commercial reference compounds (Sigma-Aldrich), by comparison of mass spectrum and RI with those described on the web [24] and by comparison of their mass spectra with those contained in the Wiley library. Results from the volatile analysis are given in area units (AU).

### 3.5. Statistical Analysis

The effect of cooking time (5 h vs. 12 h), cooking temperature (60 °C *vs.* 80 °C) and vacuum packaging (vacuum packaging *vs.* air packaging) was analyzed by a three-way analysis of variance together with their interaction (time × temperature × vacuum-packaging), using the GLM procedure (SPSS 15.0, SPSS, Chicago, IL, USA). Tukey’s test was used at the 5% level to make comparisons between the means of the eight time × temperature × vacuum combinations.

## 4. Conclusions

In this work the volatile organic compound (VOC) profile of pork cheeks as affected by the cooking conditions (time, temperature and vacuum status) was studied by solid phase micro-extraction gas-chromatography-mass spectrometry. As a general rule, the VOCs originating from lipid degradation were positively affected by the cooking temperature and negatively by the cooking time, while VOCs originated from amino acids and Maillard reactions were positively affected by both factors, with a scarce effect of the vacuum status. These results suggest that the combination of long cooking times and moderately high temperatures stimulates the formation of VOCs from amino acid-involved reactions, with desirable flavor meaty and roast notes, in detriment of VOCs originated from the fatty acids degradation, usually associated with off-flavors in meat.
